# Synergistic Effect of Marble Powder and Green Sand on the Mechanical Properties of Metakaolin-Cement Concrete

**DOI:** 10.3390/ma12030476

**Published:** 2019-02-04

**Authors:** Sakthieswaran Natarajan, Priyanka Murugesan

**Affiliations:** 1 Department of Civil Engineering, Anna University Regional Campus Tirunelveli, Tamilnadu 627007, India; 2 Department of Civil Engineering, Satyam College of Engineering and Technology, Kanyakumari, Tamilnadu 629301, India; mpriyanka2091@gmail.com

**Keywords:** metakaolin, marble powder, green sand, mechanical properties, microstructure study

## Abstract

The aim of this paper is to study experimentally the effect of marble powder and green sand as partial substitute for fine aggregate on the strength and durability of M40 grade concrete. The use of metakaolin as a pozzolanic admixture by using as binder replacement is also studied to assess the properties with respect to fresh and hardened state. Several formulations were prepared with constant water-binder ratio 0.4 and varying percentages of marble powder and green sand. The results indicated that the properties of concrete were much enhanced by extent incorporation of marble powder and green sand as fine aggregate and metakaolin for cement when compared to normal concrete. The microscopic studies also confirmed the viability of using green sand and marble powder as fine aggregates.

## 1. Introduction

Recent decades have witnessed the rapid demand for river sand as fine aggregate which is one of the most essential ingredients in the production of concrete [1]. Modern construction industry tries to eliminate this large demand by probing the usage of secondary materials as fine aggregate [2]. Sustainable construction requires the use of supplementary cementitious materials in the concrete as well as minimize the demand of river sand in concrete [3,4]. The high priority materials are predominantly silicate based with minimal cost majority of which are from industrial waste [5,6]. Supplementary cementitious materials are now an integral part of the concrete system to produce high strength concrete with improved durability [7,8]. Even minute substitution of cement using supplementary cementitious materials can contribute significantly towards the reduction of emission of greenhouse gases thereby making the concrete an eco-friendly material [9,10,11,12]. The waste generation from the mining process have yielded plenty of marble powder which is causing a greater environmental problem due to the high cost associated with their disposal [13]. Apart from the economic benefits the technical importance lies in the performance improvement of concrete when the by-products and wastes are used as concrete ingredients [14]. Marble powder as fine aggregate replacement provided several benefits in terms of strength enhancement as well as positive cost efficiency ratio [15]. Several studies have been done to reuse the waste marble powder so that they can be converted into an economical construction material [16]. Green sand used for metal castings. The high quality silica content present in the green sand foundries minimizes the exploitation of river sand at the same time meeting the environmental and design standards. Introducing foundry sand for river sand in concrete industry reduces the volume of dumping waste. These are here referred to as green sand or used foundries or foundry sand where the casting industries use green sand type as the source. Significant improvements in the energy and performance of the concrete have also been observed by using green sand as alternative for river sand.

Previous research works have previously investigated the effects of using foundry sand [17,18,19] and marble powder as fine aggregate replacement and have achieved significant improvements in the concrete strength [20,21]. In contrast there are only a limited number of studies available on the utilization of foundry sand in combination with other industrial by products in the concrete production and the data available is insufficient to confirm their suitability as fine aggregate replacements. Though the marble powder and foundry sand yield positive results similar to conventional fine aggregates their usage is still confined for practical applications due to the lack of practical information to the designers.

Hence the value of the present study lies on the fulfilling of the practical design requirements such as strength and durability of concrete mixes manufactured using replacements of fine aggregate by both marble powder and green sand. In addition the synergistic effect of marble powder and green sand together with a pozzolanic admixture (metakaolin) on the concrete properties addressed in detail thereby not only saving the exhaustion of natural aggregates but also reducing the efficiency of using industrial wastes as fine aggregate replacements.

The main objective of this research is to investigate the feasibility of using marble powder and green sand as fine aggregate in concrete production. The effect of metakaolin replacement in cement is also investigated. The mechanical properties of the hardened concrete made of green sand and marble powder were studied at various ages. The microstructure and hydration products were also investigated to assess the impact of using green sand on marble powder as partial replacement for fine aggregate.

## 2. Materials and Methodology

Ordinary Portland cement (OPC) 43 grade conforming to Indian standard BIS 8112-1989 [22] is used as the binder. Natural river sand conforming to zone 2 that having fineness modulus of grain gradation lies in 2.36–2.49 range is used as fine aggregate as per BIS 383-1970 [23]. Naphthalene based superplasticizer were used at a constant percentage of 2% by weight of cement. Metakaolin (MK), Marble powder (MP) and green sand (GS) were obtained from the nearby commercial agents. The chemical composition of the concrete ingredients as obtained from X-Ray Flourescence (XRF) results are shown in Table 1.

**Figure 1:**
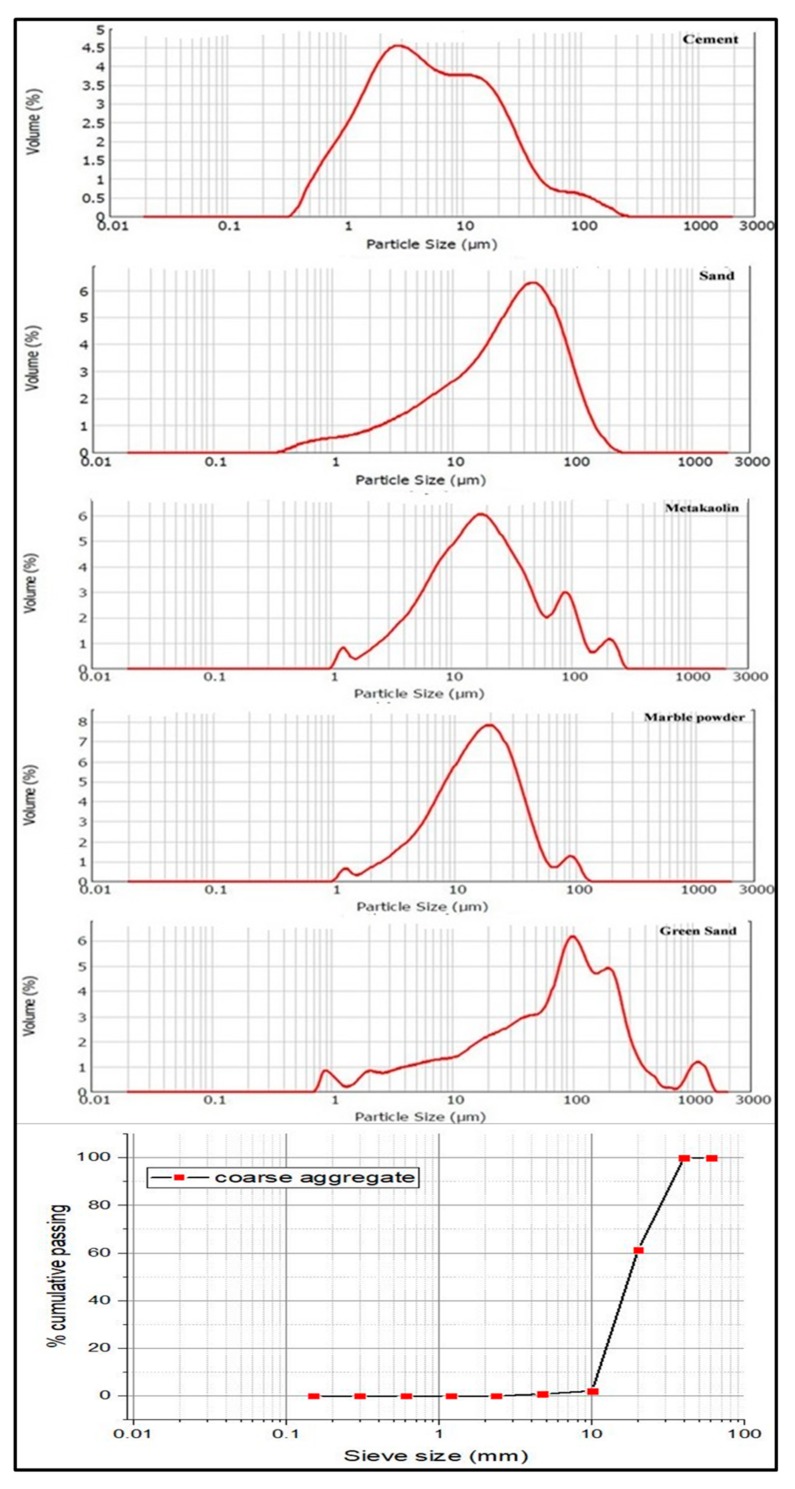
Particle size distribution of materials used in concrete.

The concrete mixes were manufactured in the proportion of 1:2.08:3.57 corresponding for Binder: Fine aggregate: Coarse aggregate, as per BIS 10262-2009 [24] referring to guidelines for concrete design mix proportioning with constant water binder ratio 0.4 to yield M40 grade of concrete mixes. Four additional concrete mixes were produced by proportioning marble powder and green sand as various percentages are shown in Table 2. The particle size of the materials observed through sieve analysis is shown in Figure 1.

The fresh state properties of the concrete mixes were determined using slump test conforming to IS1199-1959 and the hardened state properties were analyzed by conducting compressive strength on 150 mm concrete cube specimens, flexural strength test on 100 mm × 100 mm × 500 mm prism specimens and split tensile strength test on 150 mm diameter × 300 mm height cylindrical specimens at several ages conforming to BIS516-1959 [25]. Impact test (drop weight method) on 150 mm diameter and 63.5 mm height cylindrical disc specimens conforming to ACI 544 following that the specimen is place in the base plate above which a steel ball of 63.5 mm diameter is setup and loaded by dropping a hammer of weight 4.54 kg from 457 mm vertical height and static energy calculated as product of dropped mass, height of drop, number of blows at failure and acceleration due gravity. Finally the water absorption and porosity of the concrete specimens were determined according to ASTM C642-06 [26] on cubic specimens of 100 mm size that are to be normally water cured and then oven dried at 110 °C for 24 h. Water absorption is calculated with weight of specimens before and after drying. 100 mm cube Specimens are placed in boiled water for 5 h and allowed for natural loss of heat, followed with surface dry with cloth and the porosity is calculated in percentage of ratio of difference between surface dried mass and specimen mass before immersion to the difference between surface dried mass and submerged weight of the specimen. X-Ray Diffraction (XRD) analysis was done to investigate the crystallinity of the formed hydration products. The X-ray patterns of the powdered concrete mixes were recorded using Xpert Pro Panalytical diffractometer (Xpert Pro Panalytical diffractometer, supplied by PANalytical India, Haryana, India) and the obtained patterns were compared to the XRD patterns of JCPDS (Joint Committee on Powder Diffraction Studies) to analyze the mineralogical composition. The XRD patterns were recorded at 2-theta angles ranging from 10° to 90° at a step size of 0.02. The Scanning Electron Microscope (Zeiss, Shimadzu, Japan) operating at an accelerating voltage of 20 kV with secondary electron (SE) mode was used to obtain the micrographs of the concrete mixes. The SEM examination was done on small fragments of size 5mm obtained from the concrete specimens after 28 days of curing.

## 3. Results and Discussion

### 3.1. Workability-Slump

**Figure 2:**
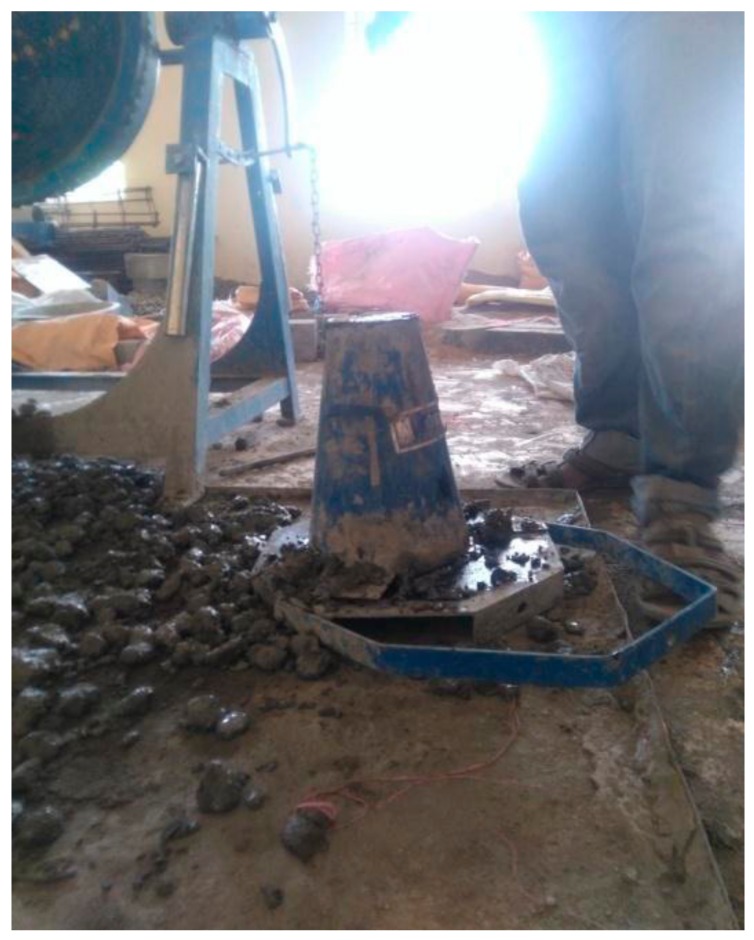
Slump cone test on concrete mixes.

**Figure 3:**
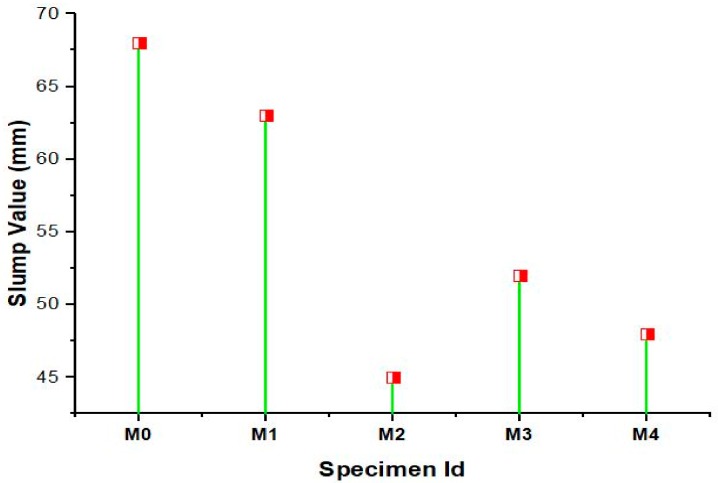
Slump values of various concrete mixes.

The workability of the concrete mixesis conducted as shown in Figure 2 and observations made by the slump test are shown in Figure 3. The workability was considerably decreased for all the mixes with metakaolin as cement replacement and marble and green sand as fine aggregate replacement. However the concrete mixes suffered from variations in workability due to the grading and shape of the fine aggregates and the characteristics of the materials used as fine aggregate. The reduction may also be due to the enhanced friction exhibited by the green sand and marble powder that are relatively more angular and rougher in texture when compared to that of conventional river sand. The decrease in workability with increasing green sand content may be probably due to the presence of clay type material which effectively decreases the fluidity of the fresh concrete. The workability results were in agreement to the previously established results which showed that the green sand substitution up to 10% do not cause a considerable change in the workability [21]. The workability of the concrete is also directly proportional to the fineness of the materials used in concrete and hence the increase fineness of the green sand increases the water demand of the concrete which consequently reduces the workability of the concrete. In addition the higher fineness of the green sand increases the surface of the formed hydrated products which leads to absorb more water by which the workability is decreased as a result. Though marble powder absorbs less water than the normal river sand the workability of concrete was found to decrease with increase in marble powder content. Studies have shown that the workability of the concrete mixes are negatively influenced by the marble powder substitution and this effect gets pronounced at greater substitution levels beyond 15% [16]. Thus the workability of the concrete can be negatively correlated with the green sand and marble powder content. Moreover the decreased workability of the concrete mixes may also be attributed to the cement replacement by metakaolin. The property of agglomeration and compounding effect of metakaolin causes a negative influence on the workability of the concrete. This negative effect of metakaolin exhibited on the workability of concrete is mainly due to the high specific surface area and pozzolanic effect that increases the wettable surface area. This increase in the wettable surface area requires more water which leads to reduction in workability. The flocculation caused by electrostatic attraction between the cement and metakaolin particles decreases the workability by minimizing the slump values.

### 3.2. Compressive Strength

**Figure 4:**
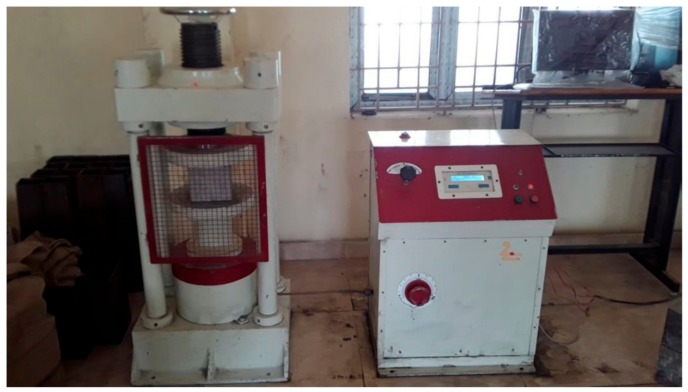
Compression Testing Machine.

**Figure 5:**
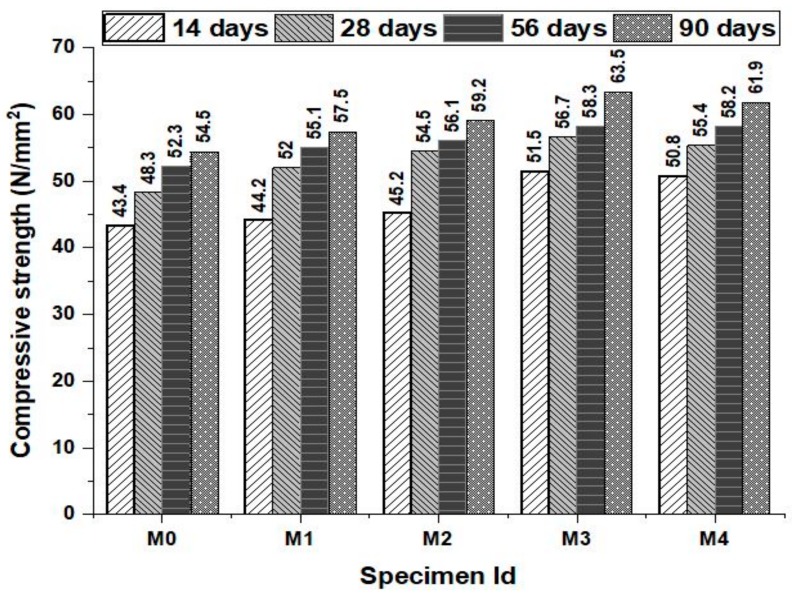
Compressive strength of the concrete mixes at various ages.

Figure 4 shows the test setup (Scientific, 300 ton capacity, supplied by Shiv Ganga Industries, India) and the compressive strength of the concrete mixes at the ages of 14, 28, 56 and 90 days is shown in Figure 5. It can be clearly inferred that the compressive strength of the concrete increased with increase in substitution rate of green sand and marble powder as fine aggregate in a concrete. The improvement in the compressive strength of the concrete is due to the presence of more silica content that enhanced the formation of hydration products in the concrete. The compressive strength of the concrete specimens is also influenced by the percentage of metakaolin and it can be observed that the addition of metakaolin improved the compressive strength when used as a partial substitute for cement and this can be attributed to the property of metakaolin such as the filler effect, pozzolanic reaction of metakaolin with calcium hydrate and the compounding effect of metakaolin. The very early strength is also due to accelerated hydration forming aluminum ions during dehydroxylation. In the presence of water, Calcium hydrates and crystalline products of aluminate hydrate and alumina-silica hydrates promoting pozzolanic activation depending on kaolin to hydrates. Metakaolin was set to use 5% throughout for all mixes that lies within the remarkable limits from the earlier studies. Metakaolin substitution reduces the continuity of pores that make the matrix less permeable. The increase in strength was observed that the mixes performed well at the early strength and this may due to the contribution of metakaolin to produce the paste with less pores. The results observed at 90days also showed that the mix with 15% of green sand and marble powder performed well than all other mixes. The marble powder substitution also contributed to the strength of the concrete by their filler effect that densify the paste structure of the concrete by using reducing the pore volume of the concrete. It can also be seen that the compressive strength of the concrete increase up to 15% replacement of the fine aggregate beyond which slight reduction in the compressive strength of the concrete was observed. The reduction in the compressive strength beyond 15% replacement of the fine aggregate by green sand and marble powder may be probably due to the increased surface area of the fine aggregates but led to the reduction in the water cement gel in the matrix. This reduced cement gel is not sufficient to bind the fine aggregates and coarse aggregates that affect the structural integrity of the concrete accompanied by slight reduction in their compressive strengths. Moreover the interruptions on the grain size distribution caused by these powder filler materials reduce the compressive e strength of the concrete by increasing the pore spaces in the concrete. This is in agreement to the similar study conducted using green sand as fine aggregate replacement beyond 20% also showed reduced compressive strength due to the presence of more amount of silica that significantly affected the hydration process [21]. The compressive strength increment may also be caused due to the metakaolin substitution due to densification of the microstructure of the concrete [9].

### 3.3. Flexural Strength

**Figure 6:**
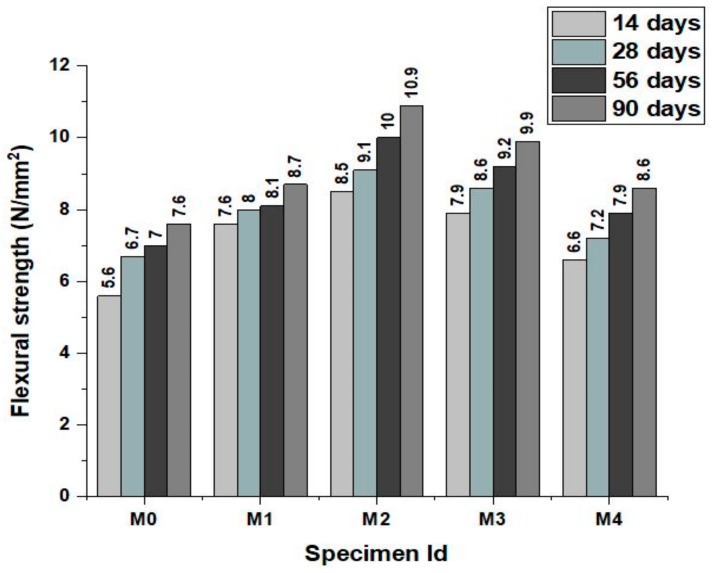
Flexural strength of the concrete mixes at various ages.

**Figure 7:**
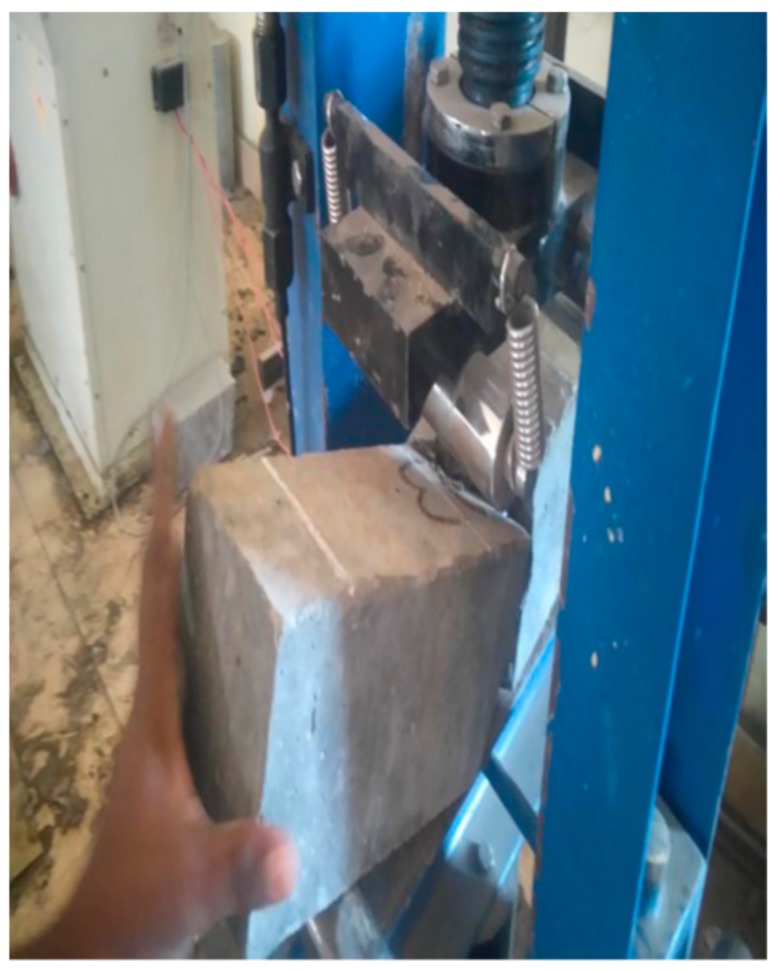
Flexural strength Test setup.

The flexural strength development of the concrete mixes at various ages of 14, 28, 56 and 90 days are shown in Figure 6 and failure of specimen under testing (Scientific, supplied by Shiv Ganga Industries, India) is shown in Figure 7. The results clearly explain the influence of green sand and marble powder in improving the flexural strength at all ages of the concrete when compared to the normal concrete. This improvement in the flexural strength of the concrete mixes may be attributed to the higher fineness and morphologically rough texture of the marble powder and green sand in comparison to river sand. The fineness of these powdered materials densified the cement matrix and the rough surface texture improved the adhesion in the concrete that actively bonded the cement and the aggregates. The declining trend in the flexural strength may be due to the fact that after the attainment of the optimum content of replacement the increased substitution of the fine aggregate by marble powder and green sand loses its effectiveness as a filler agent by increasing the specific surface area of the concrete.

### 3.4. Split Tensile Strength

**Figure 8:**
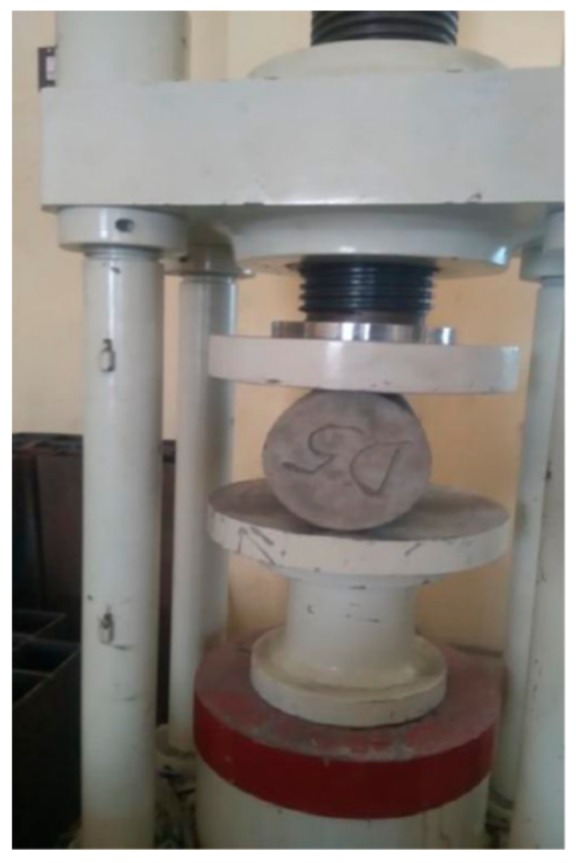
Split tensile strength test setup.

**Figure 9:**
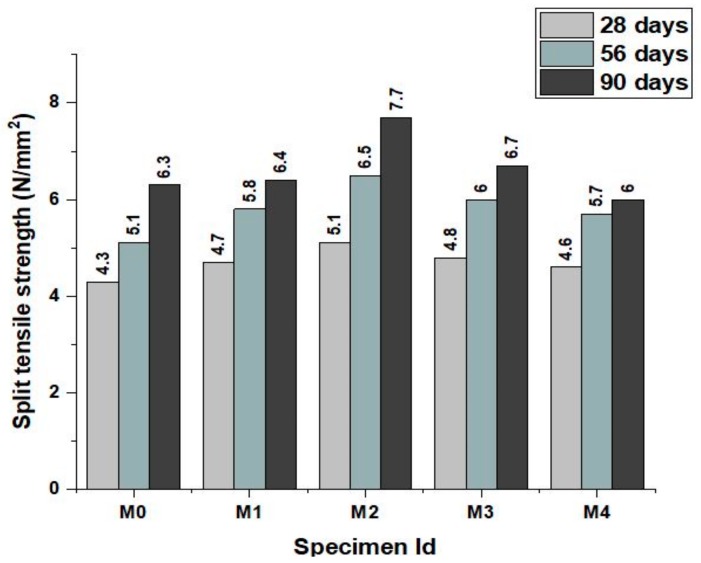
Split tensile strength of the concrete mixes at various ages.

The splitting tensile strength test (Scientific, supplied by Shiv Ganga Industries, India) setup on cylinder specimen is shown in Figure 8 and that of the concrete mixes at various ages is shown in Figure 9. The improvement in the split tensile strength of the concrete follows the same trend of the flexural strength improvements. It can be observed that the splitting tensile strength of the concrete increases with the increasing percentage level of the fine aggregate by green sand and marble powder. This improvement in the splitting tensile strength may be due to the strong bonding behavior exhibited by the marble powder and green sand replacement and also due to the pozzolanic effect of metakaolin that increases the denseness and compactness of the concrete. This shows that the study of flexural and split tensile strength was about to be similar conforming that the optimum replacement levels for both fines and binder with marble, green sand and metakaolin to be limit with 10% and 5%respectively. Beyond 20% replacement of fines, the mixes performed good than control but the trend of negative linear relation with increase in replacement percentage. It is also observed that increase in strength with age is close to the conventional concrete but the early strength at 28 days was clearly understood to be due the densification of concrete by metakaolin. It is also understood that the water requirement needs to be corrected and addition of superplasticizers is strongly recommended for the mixes beyond 30% replacement. Metakaolin contributes towards the hydration of paste and early strength of concrete. Performance of mixes at the age of 90 days was observed to be right linear upto 20% replacement and thereafter the declining trend and then for the mix M4, the split tensile strength seems the same that of control concrete. This agrees well to the previously conducted studies on metakaolin as cement replacement [8].

### 3.5. Impact Strength

**Figure 10:**
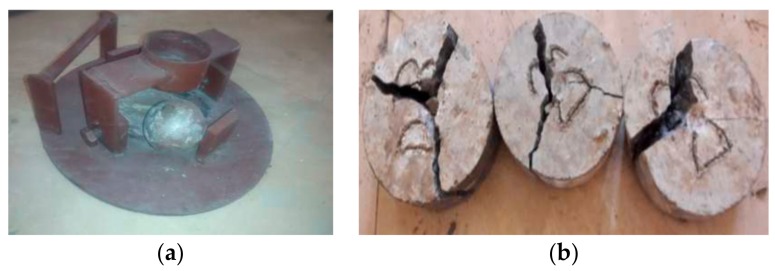
Impact strength Apparatus and specimens. (**a**) base plate and steel ball (**b**) specimen subjected to impact test.

**Figure 11:**
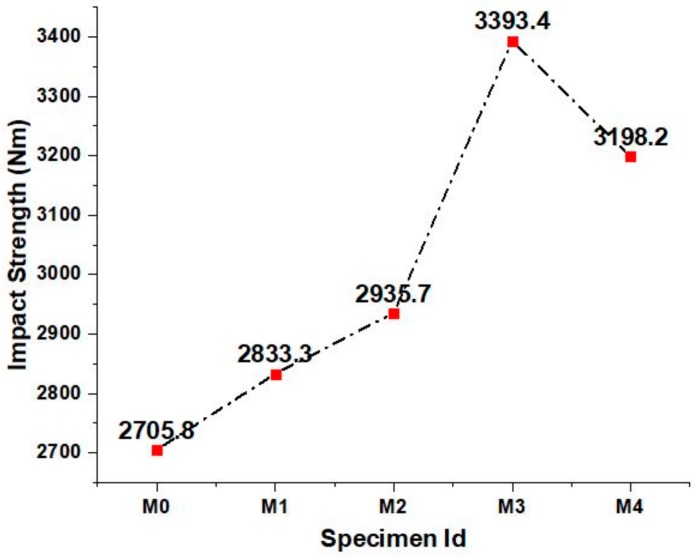
Impact strength of various concrete mixes.

The impact strength apparatus and specimens at failure is shown in Figure 10 and that of the concrete mixes is shown in Figure 11. It is observed that the impact strength of the concrete increased with increasing fine aggregate replacement up to a certain extent. This shows the higher energy absorbing capacity of the concrete due to the fine aggregate replacement. The higher fineness of the marble powder and green sand filled the interstitial spaces between the aggregates and the cement paste that led to the increased impact energy of the concrete. Moreover the addition of metakaolin as partial cement replacement also influenced the impact strength to the greater extent by the formation of more stable reaction products which are relatively stronger than that formed in the normal concrete. The declining trend in the impact strength of the concrete may be due to the increase in the heterogeneity caused by the marble powder and green sand replacement that increases the brittleness in the concrete thereby by reducing the cohesion in the concrete.

### 3.6. Water Absorption and Porosity

**Figure 12:**
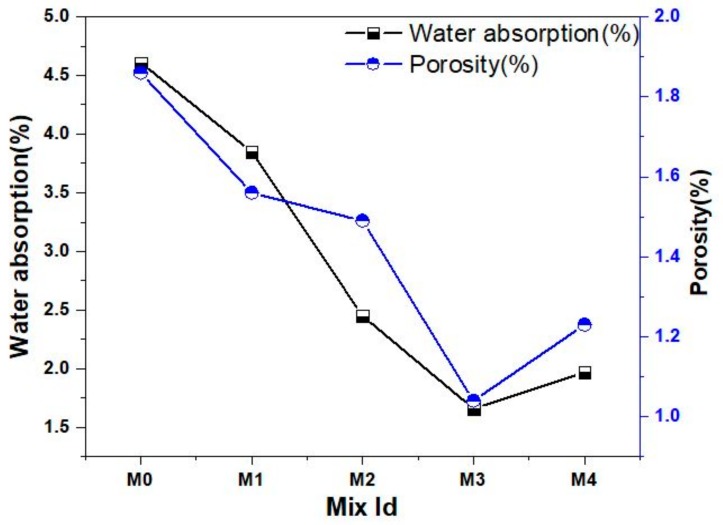
Water absorption and porosity of various concrete mixes.

The water absorption values of the concrete mixes with and without the fine aggregate replacement are provided in Figure 12. It is observed that the water absorption of the concrete decrease with increase in the percentage of replacement in the fine aggregate by marble powder and green sand. This decreased water absorption behavior of the concrete may be explained by the reduction in the voids caused by the green sand and marble powder. The lower water absorption capacity of the concrete mixes containing marble powder and green sand can be related to the nature of the pore spaces within the hardened concrete. The water absorption of the concrete is mainly dependent on the cement paste which is the only continuous phase in the concrete. The aggregates usually contain discontinuous pores that are enveloped by the continuous phase of cement paste that causes reduction in the pore and also the water absorption of the concrete. Hence the influence of the aggregate is very small and do not contribute much to the water absorption of the concrete. Hence the quality of the cement matrix has the greatest effect on the water absorption behaviour of the fully compacted and hardened concrete. In this present study the use of metakaolin as partial replacement for cement improves the pore structure of the concrete by reducing the total pore volume of the concrete by the formation of denser hydration products during the period of curing. Figure 12 also shows the change in the porosity values of the concrete mixes using green sand and marble powder as partial substitute for fine aggregate. From Figure 12, it can be seen that the porosity of the concrete decreases with increase in the percentage of marble powder and green sand substitution. The improvement in the pore structure of the concrete could be explained as a result of the filler effect of the green sand and marble powder. Moreover the porosity of the concrete was also much influenced by the addition of metakaolin. The micro filling effect and the pozzolanic reaction of the metakaolin particle highly refined the pores by enhancing the micro and macro structures of the cement paste. The increase in the porosity values may be attributed to the non-uniform grading caused by these powdered aggregates when used with metakaolin that possesses a more specific surface area than that of cement, leading to the increased absorption of water, as evident from previous research [8,9].

### 3.7. X-Ray Diffractometer

**Figure 13:**
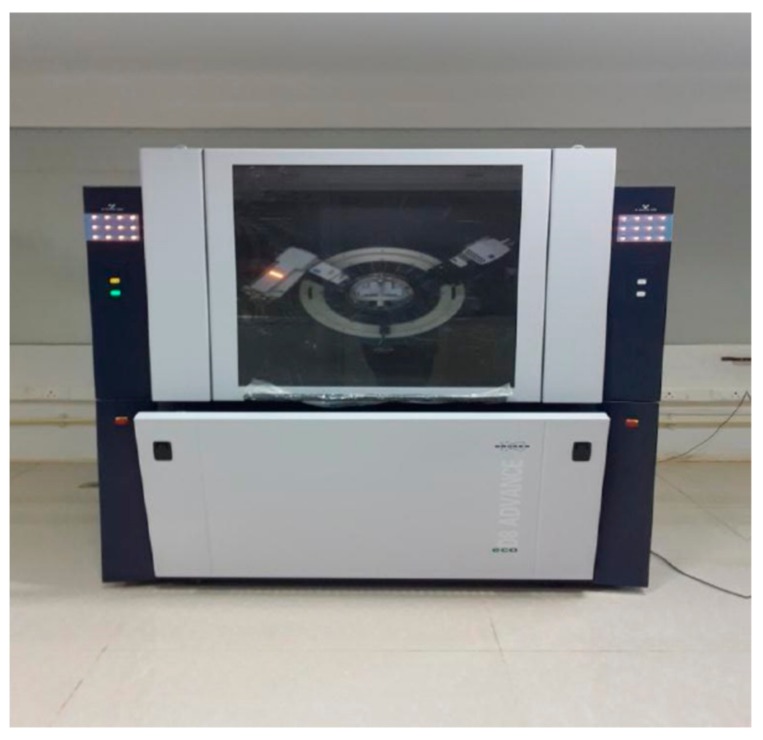
X-Ray Diffractometer.

**Figure 14:**
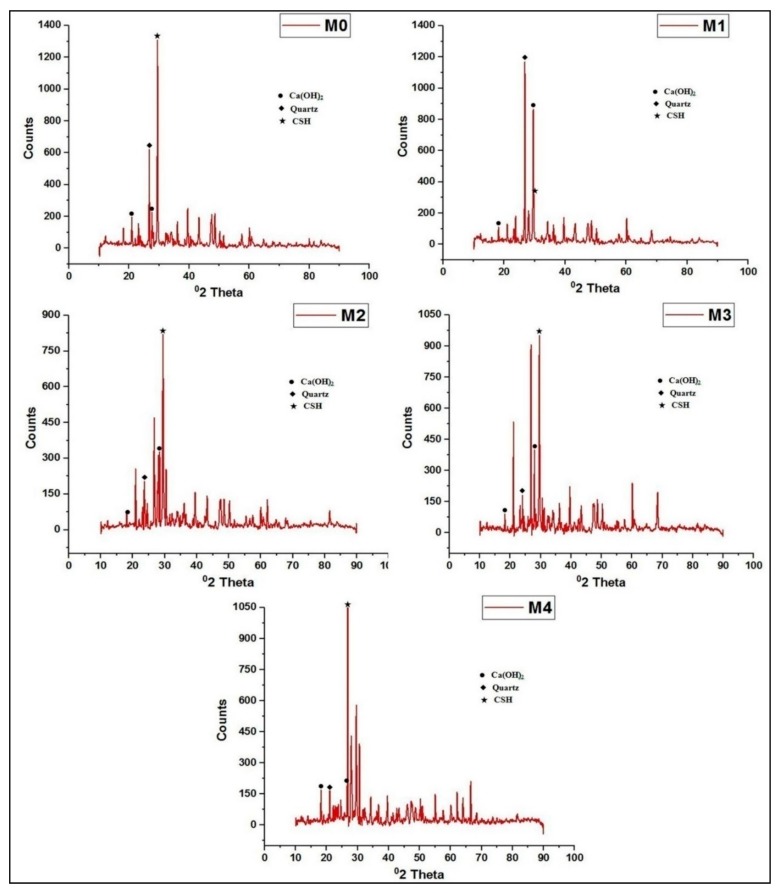
XRD patterns of various concrete mixes.

The XRD studies (Figure 13) done to analyze the components of the concrete mixes and the obtained diffraction patterns are shown in Figure 14. The main peaks that were formed were identified as quartz, portlandite Calcium Hydroxide and Calcium Silicate Hydrate gel (CH and CSH). The patterns clearly indicate the strong peak of quartz indicating the presence of free silica. The hydration product phases CH and CSH were found to vary in their intensities in the patterns exhibited by the hydrated pastes. It can be clearly seen that the peaks of portlandite were found to decrease in all the concrete mixes indicating the effect of pozzolanic action of metakaolin that effectively converted the CH to CSH. The quartz peak were also found to decrease or increase in the intensity indicating the interaction of the free silica with the CH to form CSH gel. The free silica increases the intensity of the quartz peak in the diffraction patterns with increasing marble powder and green sand due to the relatively higher amount of silica present in these powdered aggregates when compared to the river sand. The formation of high intense CSH peaks with the relatively less intense CH peaks helps in improving the pore structure of the concrete which subsequently increases the strength of the concrete.

### 3.8. SEM

**Figure 15:**
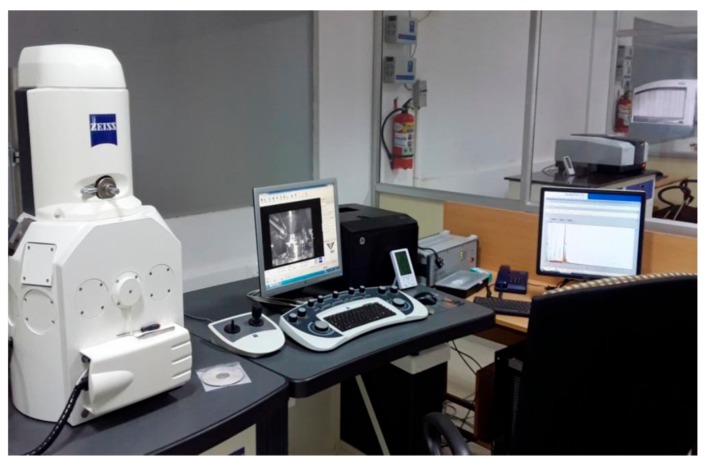
Scanning Electron Microscope.

**Figure 16:**
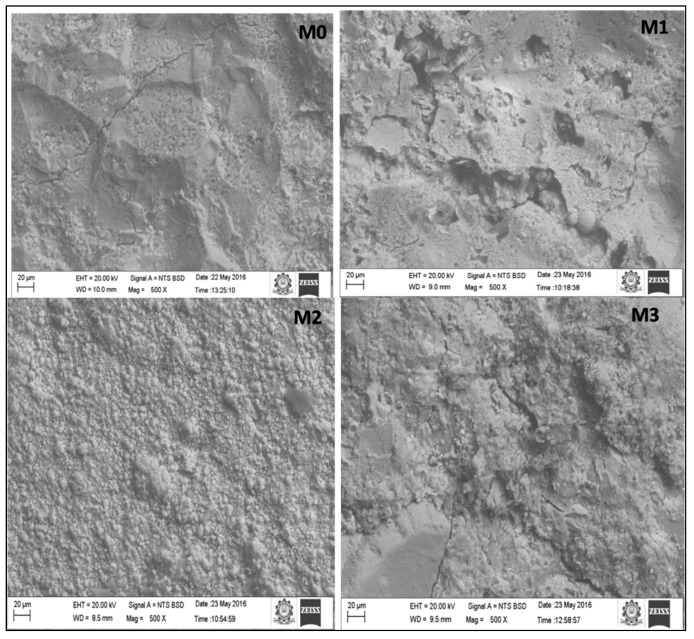

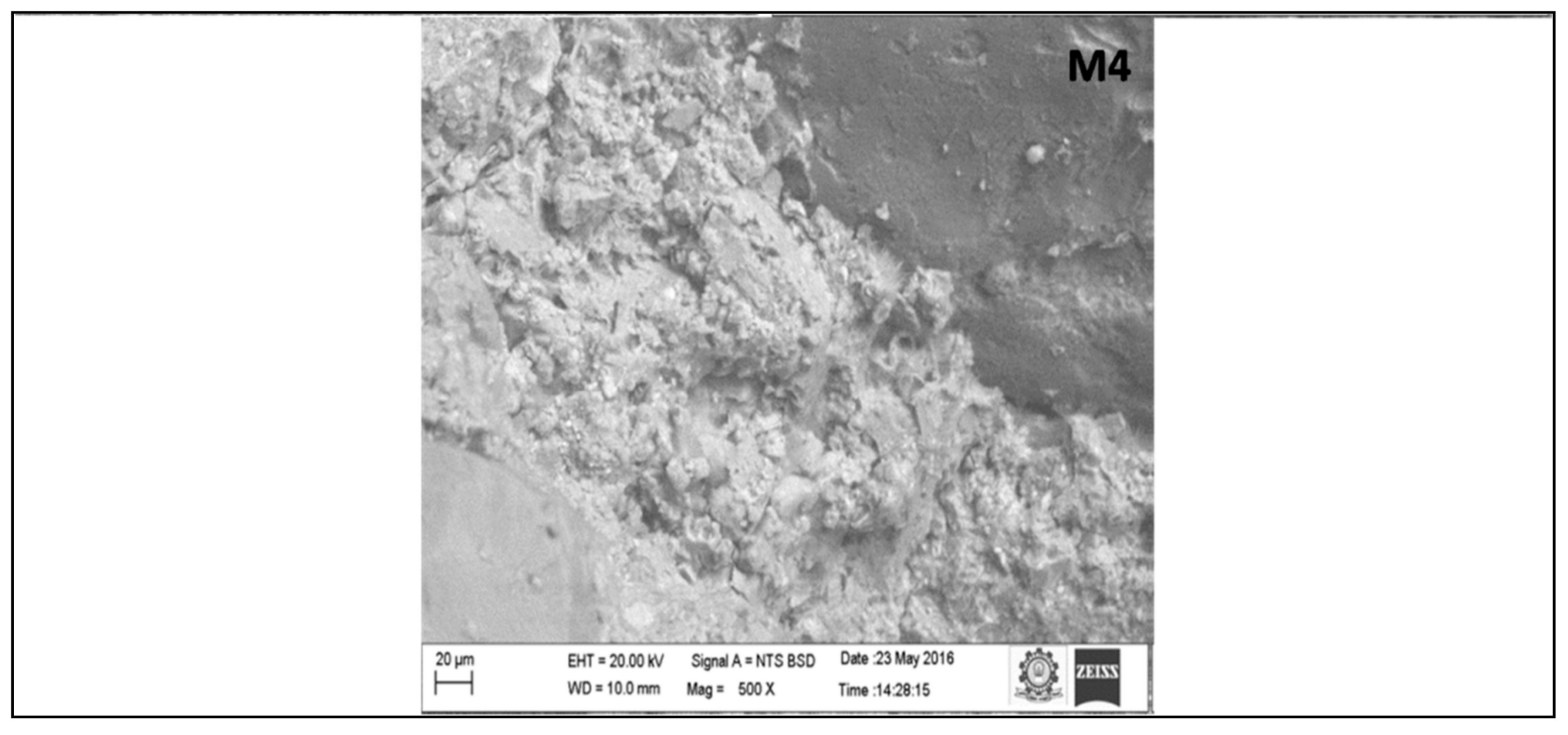
SEM images of various concrete mixes.

The morphology of the concrete mixes as obtained from the Scanning Electron Microscope Equipment (Figure 15) and result images is shown in Figure 16. The main strengthening phase present in the concrete is the CSH gel. The formation of CSH gel also depends on the grain size and distribution of the aggregates. The pore size distribution and composition of the continuous phases in the concrete also plays a vital role in the CSH gel formation. From the SEM images the bright phases of CSH gel formations are seen in the concrete mixes. It can be clearly seen that the CSH gel formed was spread uniformly throughout the concrete mixes that formed a layer over the aggregates that acted as binder for the cement matrix and the aggregates. The presence of dark regions in the SEM images indicates the voids or pores in the concrete which is visibly reduced in all the concrete mixes indicating the contribution of marble powder and green sand in porosity reduction. Since the strength of the concrete is inversely proportional to the number of voids, the improved strength of the concrete may be attributed to the reduction in voids of the concrete. The microstructure also shows the well distribution of the marble powder and green sand in the concrete that improved the morphology of the concrete by densifying the microstructure. Moreover the use of metakaolin as a partial substitute for cement also enhanced the hydration reaction, due to its pozzolanic behavior, which transforms the CH to CSH gel and fills the pores of the concrete, which in turn increases the compressive and flexural strength.

### 3.9. Regression Analysis

**Figure 17:**
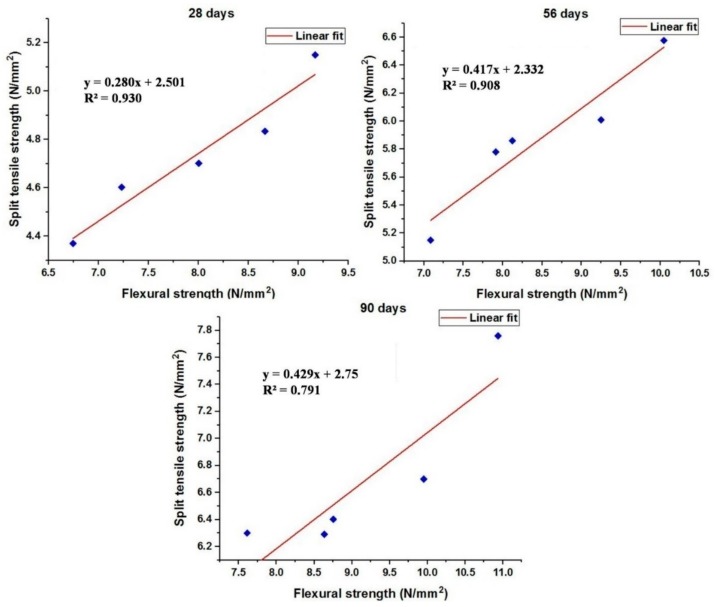
Relation between flexural strength and split tensile strength of the concrete mixes at various ages.

**Figure 18:**
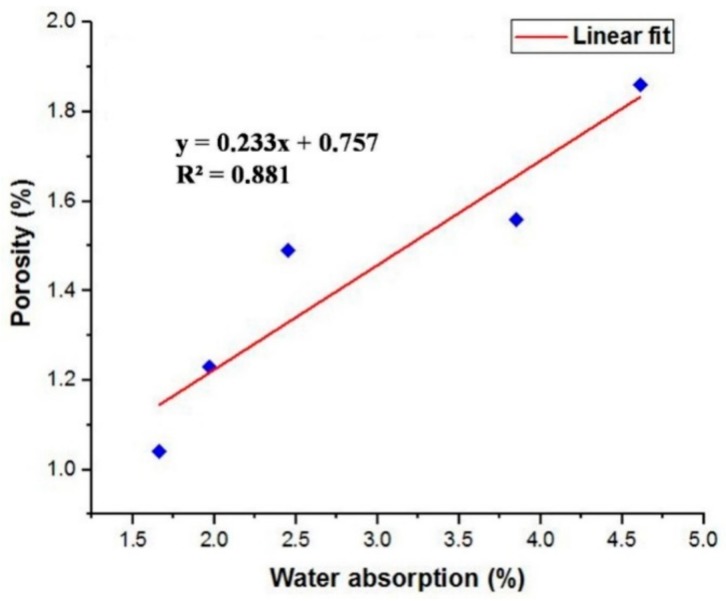
Relation between water absorption and porosity of the concrete mixes.

With the help of the experimental data, graphs were plotted between split tensile strength and flexural strength of concrete mixes at various ages. The linear relationship between split tensile strength and flexural strength is obtained through the predicted equation and their corresponding regression coefficient R^2^ values are also shown in Figure 17 and Figure 18.

## 4. Conclusions

This study mainly aims at utilizing marble powder and green sand as partial substitute for fine aggregate and metakaolin for cement. From the experimental results obtained the following conclusions can be withdrawn.

1. The workability of the concrete was very much influenced due to the addition of green sand and marble powder. The relative decrease in the workability was achieved due to the increase in amount of water required by the aggregates and the normal sand. Moreover decrease in the workability may also be caused due agglomeration and compounding effect exhibited by the metakaolin particles.
2. An increase in the compressive strength of all the mixes occurred at various ages. The filler effect and the pozzolanic reaction of metakaolin with calcium hydrate also contributed to the increase in the strength of the concrete. This enables the use of marble and green sand as construction materials by minimizing the utilization of the river sand as fine aggregate and the reduced production of cement by replacing with metakaolin.
3. The flexural strength of the concrete mixes was enhanced due to the fine aggregate replacement and metakaolin replacement. The use of marble powder and green sand within 15% was found to be optimum to increase the flexural strength of the concrete when used in combination with metakaolin as cement substitute.
4. The splitting tensile of the concrete was also improved up to 15% beyond which the decrease in the reduction in the split tensile strength was observed. This shows that using 15% green sand and marble powder was found to be adequate as far as the strength parameters are concerned.
5. The impact strength of the concrete was much enhanced for the concrete containing metakaolin as cement replacement and marble powder and green sand as fine aggregate replacement that proved to be efficient in absorbing the impact energy acting on the concrete.
6. The use of marble powder and green sand as fine aggregate replacement created a favorable effect on reducing the porosity of the concrete. The reduction in the water absorption caused a decrease in the voids and is responsible for the improvement in the mechanical strength and impermeability properties of concrete. The addition of metakaolin caused stronger bonding of the cement paste and strengthens the adherence of the cement and the aggregates by the formation of CSH gel which is also responsible for the reduction in the porosity values.
7. The X-ray diffraction studies also showed the minimal presence of calcium hydroxide that confirmed the consumption of calcium hydroxide in the hydration reaction which is responsible for the dense microstructure of the concrete by the addition of CSH gel. The reduced calcium hydroxide peaks inherits the formation of CSH gel that is responsible for the improved strength and durability of the concrete.
8. The SEM images of the concrete also showed reduction in the voids in the concrete and the well distributed CSH gel that effectively bonded the fine aggregates with the cement matrix. The SEM micrographs are coherent for the obtained mechanical properties and the morphology of concrete mix and shows that the marble powder and green sand in combination with metakaolin can be used for making good quality concrete with higher strength.
9. The prediction models obtained from the experimental data showed the relationship between various strength parameters of the concrete and the obtained R^2^ values indicate the conformity of the interrelationship between the variables.

The final conclusion can be withdrawn that when green sand and marble powder are used as fine aggregate replacement and metakaolin as partial substitute for cement the concrete mixture with high strength properties compared to that of the normal concrete can be obtained. The incorporation of these powdered fine aggregates instead of normal river sand proves to be beneficial to obtain concrete with higher strength. Moreover this type of concrete produced is also environment friendly and economically feasible by large utilization of the waste marble powder and green sand.

## Figures and Tables

**Table 1:** Chemical composition of the cement, marble powder, green sand and metakaolin.

Component	OPC (%)	MP (%)	GS (%)	MK (%)
SiO_2_	20.85	0.79	87.91	53.75
Al_2_O_3_	4.78	0.21	4.70	43.82
Fe_2_O_3_	3.51	0.06	0.94	0.45
CaO	63.06	55.42	0.14	0.16
MgO	2.32	0.25	0.30	0.00
SO_3_	2.48	0.24	0.09	0.02
K_2_O	0.55	0.02	0.25	0.18
Na_2_O	0.24	0.10	0.19	0.26
TiO_2_	0.25	0.00	0.15	0.86
Mn_2_O_3_	0.05	0.00	0.02	0.00
Cl	0.01	0.07	0.00	0.00

Ordinary Portland cement (OPC), Marble powder (MP), green sand (GS) and Metakaolin (MK).

**Table 2:** Level of Replacement in binder and aggregates.

Mix ID	Binder		Fine Aggregate			Coarse Aggregate
	Cement	Metakaolin	Sand	Green Sand	Marble Powder	
M0	100%	0%	100%	0%	0%	100%
M1	95%	5%	90%	5%	5%	100%
M2	95%	5%	80%	10%	10%	100%
M3	95%	5%	70%	15%	15%	100%
M4	95%	5%	60%	20%	20%	100%
